# Gene expression profiles of beta-adrenergic receptors in canine vascular tumors: a preliminary study

**DOI:** 10.1186/s12917-022-03317-1

**Published:** 2022-05-30

**Authors:** Jordi Clanxet, Mariana Teles, Javier Hernández-Losa, Manuel Ruiz-Echarri Rueda, Luis Benitez-Fusté, Josep Pastor

**Affiliations:** 1 Department of Animal Medicine and Surgery, Universitat Autònoma de Barcelona, 08193 Barcelona, Spain; 2 Department of Cell Biology, Physiology and Immunology, Universitat Autònoma de Barcelona, 08193 Barcelona, Spain; 3 Institute of Biotechnology and Biomedicine, Universitat Autònoma de Barcelona, 08193 Barcelona, Spain; 4 Molecular Biology Laboratory, Department of Pathology, Hospital Universitario Vall d’Hebron, Passeig Vall d´Hebron, 119-129, 08035 Barcelona, Spain; 5 Bristol Royal Infirmary, Marlborough Street, Bristol, BS2 8HW UK; 6 Hospital Veterinari del Mar, C/ Marina 69, 08005 Barcelona, Spain

**Keywords:** ADBR, Beta receptors, Dog, Hamartoma, Hemangiosarcoma, Hemangioma

## Abstract

Beta adrenergic receptors (β-AR) play a key role in regulating several hallmark pathways of both benign and malignant human and canine tumors. There is scarce information on the expression of β-AR in canine vascular tumors. Therefore, the purpose of the present research work was to study the mRNA expression levels of the three subtypes of the β-AR genes (*ADRB1, ADRB2, ADRB3*) in hemangiosarcoma (HSA) and hemangioma (HA), as well as in vascular hamartomas (VH) from dogs.

Fifty samples (*n* = 50) were obtained from 38 dogs. Twenty-three animals had HSA, eight animals HA and seven animals VH. HSA were auricular (*n* = 8), splenic (*n* = 5), cutaneous (*n* = 6), auricular and splenic (*n* = 2), cutaneous-muscular (*n* = 1) and disseminated (*n* = 1). There were seven cases of HSA that were divided into primary tumor and secondary (metastatic) tumor. Skin and muscle samples with a normal histological study were used as control group. *ADRB* gene expression was determinate in all samples by real-time quantitative PCR. Results showed that *ADRB1, ADRB2* and *ADRB3* were overexpressed in HSA when compared to the control group. *ADRB2* was overexpressed in HA when compared to the control group. HSA express higher values of *ADBR1* (*p* = 0.0178) compared to VH. There was a high inter-individual variability in the expression of the three subtypes of *ADBR*. No statistically significant difference in the expression of *ADBR* genes were observed between HSA primary when compared to metastatic or in different anatomical locations. In conclusion, canine HSA overexpress the three β-AR subtypes and canine HA β2-AR. High variability was observed in β-AR mRNA levels amongst HSA cases.

**Supplementary Information:**

The online version contains supplementary material available at 10.1186/s12917-022-03317-1.

## Introduction

Beta adrenergic receptors (β-AR) play a key role in regulating several hallmark pathways of both benign and malignant human and canine tumors [1–4]. There are three subtypes of β-AR (β1, β2 and β3) with a variable physiological distribution in tissues. The β1-AR subtype is mainly located in the heart, kidney and adipocytes; β2-AR is mainly distributed in the lungs and bronchi, vascular smooth muscle, sympathetic terminal, heart, uterus, skeletal muscle, among others; and β3-AR is found in adipocytes, uterus, bladder and heart [1, 2]. The three subtypes of β-AR have also been identified in primary and metastasis tumors of the brain, lung, liver, kidney, adrenal glands, ovary, prostate, lymphoid tissue, melanoma, bone marrow and vasculature [5, 6].

In human medicine, the β1-AR and β2-AR subtypes are involved in tumoural mechanisms, such as proliferation and angiogenesis. Recently, the β3-AR subtype has also been found to take part in these pro-tumoral processes [7]. Moreover, β2-AR has been specially described as overexpressed in human angiosarcoma. Furthermore, recent data revealed a strong correlation between the use of β-AR antagonists and a significant reduction of the cancer progression, metastasis and mortality in affected patients, even in advanced stages of the disease [4, 8, 9]. Also the therapeutic targeting of the β-AR has shown efficacy in the treatment of benign vascular tumors, such as infantile hemangiomas, with propranolol becoming the treatment of election since its serendipitous discovery [10, 11]. Thus, β-AR antagonists could be used as an adjunct drug to current therapeutic strategies in clinical oncology [5, 9, 12, 13].

Vascular tumors are a common neoplasia in dogs, being hemangioma (HA, benign) and hemangiosarcoma (HSA, malignant) the most frequent vascular tumors diagnosed [14, 15]. Canine HA are mostly located in the skin (84%) and it is widely distributed in the head, neck, trunk and extremities. HA account for approximately 4.5% of all skin neoplasms and HSA for less than 1% [16, 17]. HA has a benign behavior and complete surgical resection is usually curative [15]. Canine HSA is a highly aggressive neoplasia that is usually visceral [18]. This type of tumor is characterized by a high metastatic rate and a poor response to the current available treatment options, which leads to a poor prognosis [4, 19]. Surgery is the treatment of choice for most primary HSA. However, due to its highly aggressive behavior and the poor outcome of the surgery alone, adjuvant chemotherapy is necessary in all cases [4, 19]. HSA have been presumed to have its origin from transformed endothelial cells, although, new data suggest a pluripotent bone marrow progenitor as the cell of origin [20].

Hamartomas are unifocal/multifocal overgrowths of mature non-tumoral cells and tissue native to the organ in which it occurs. Due to their limited growth, they are considered developmental malformations [21]. Vascular hamartomas (VH) are the most frequent form of hamartomas found in domestic animals [22] and correspond to a disorganized and excessive proliferation of the vascular tissue [23].

With all the above in mind, the purpose of the present research work was to study the mRNA expression levels of the three subtypes of the β-AR genes (*ADRB1, ADRB2, ADRB3*) in canine vascular tumors (HA and HSA), as well as in VH.

## Materials and methods

### Criteria for case inclusion

Samples from biopsies and necropsies from the Diagnostic Service of Veterinary Pathology of the Universitat Autònoma de Barcelona (UAB) were retrospectively selected. All dogs were sampled with the owner’s permission for clinical or diagnostic reasons, and an owner informed consent was obtained in order to use those samples for future studies. Inclusion criteria was a formalin-fixed and paraffin embedded tissue sample with a histological diagnosis of HA, HSA or VH. Both primary and metastatic HSA were included in the study. Primary and secondary tumors were classified based on the clinical record and followed up of the case, in cases where followed up were not available the larger tumor was considered the primary specially if they affect the auricle or the spleen As a control group, skin and muscle samples with a normal histological diagnosis were used. Control samples were processed in the same way that tumor samples.

### RNA extraction, cDNA synthesis and β-adrenergic receptors (ADRB) gene expression

Only paraffin conserved samples with more than 800% of tumor sample confirmed by histology were used. Paraffin conserved tissue samples were numerated from 1 to 50 in order to have each case identified. For some animals a pool of tumors in different localization was used to extract RNA as it is stated in table 1 at the supplementary material). After deparaffinization of 10 µm paraffin embedded tissue samples, RNA was extracted using the High Pure FFPET RNA Isolation Kit (Roche). Cells were lysed in 100 µL RNA Tissue Lysis Buffer, 16 µL 10% SDS and 40 µL of Proteinase K during 30 min at 85 ºC and shaking at 600 rpm. After this step, 60 µL of Proteinase K were added and the tissue samples were incubated at 55 ºC and shaking at 600 rpm for 30 min.

After cell lysis, the samples were incubated during 15 min at 15 to 25 ºC with the DNAase working solution to enhance the RNA extraction. After this, the samples were centrifuged several times to eliminate debris and, finally, 50 µL of RNA Elution Buffer was added in order to obtain the pure total RNA. RNA quantification quantity (ng/µL) and the quality (260/280 ratio) was done using a NanoDrop Spectrophotometer (Thermo Fisher Scientific).

Reverse transcription (RT) was performed with 1 μg of RNA using the High-Capacity cDNA Reverse Transcription Kit (Thermo Fisher Scientific, ABI Applied Biosystems 9902 Veriti PCR Thermal Cycler). The program was 10 min at 25 ºC, 120 min at 37 ºC, 5 min at 85 ºC and forever at 4 ºC.

For the *ADRB* gene expression RT-qPCR was performed using the following primers: *ADRB1* (Biorad; Dog #ENSCAFT00000017826.4), *ADRB2* (Biorad; Dog #ENSCAFT00000029135.3), *ADRB3* (Biorad; Dog #ENSCAFT00000009965.3), *SDHA* (Biorad; Dog #ENSCAFT00000017502.3) and *GAPDH* (Biorad; Dog #ENSCAFT00000023939.3). *SDHA* and *GAPDH* were selected as housekeeping genes.

Each well contained 10 µL of 2 × SsoAdvanced™ Universal SYBR® Green, 1 µL of 20 × PrimePCR, 8 µL of nuclease-free water and 1 µL of cDNA. Control samples, nuclease-free water and six cDNA samples were included in triplicate for each primer in all the plates.

After assessing primers assembly, RT-qPCR was performed using the 7500 Fast Real-Time PCR System (Applied Biosystems) for the *ADRB* gene expression. The RT-qPCR program was: 2 min at 95 ºC (1 cycle) and 5 s at 95 ºC and 30 s at 60 ºC (40 cycles). Gene expression levels were determined using the ΔΔC_t_ method normalized to the C_t_ mean of GAPDH and SDHA. Skin and muscle tissue samples from twelve healthy dogs were used as a control group and the three subtypes of *ADRB* expression was expressed as fold changes in VH, HA and HSA. Fold changes were calculated with the formula 2^−ΔΔCt^.

### Statistical analysis

The expression of *ADBR* genes amongst vascular tumors were studied using a non -parametric ANOVA test with Dunn’s multiple comparison test. Mann Whitney test was used to compare *ADBR* expression in primary and secondary HSA. Finally, we studied the *ADBR* expression depending on HSA tissue localization with Kruskall-Wallis test. Significance was set at *p* < 0.05 in all cases and tests were carried out with GraphPad Prism 8 (GraphPad Software, San Diego, CA, USA).

## Results

### Tissue samples

Fifty samples (*n* = 50) were obtained from 38 dogs, twenty-four were males and fourteen were females (Supplementary material Table 1). The most affected breeds were German Shepherd (one HA, nine HSA and one VH), Crossbreed (two HA and six HSA), Boxer (three HA and one VH) and Labrador Retriever (one HA and one HSA). Most animals were over eight years old in the two groups of animals with types of tumors and in the group of animals with the vascular malformation (*n* = 27; 71%). The mean age of the animals for each condition was: 8.2 years old for VH, 8.7 years old for HA and 9.7 years old for HSA. There were, eight animals with HA, twenty-three animals with HSA and seven animals with VH. HSA were auricular (*n* = 8), splenic (*n* = 5), cutaneous (*n* = 6), auricular and splenic (*n* = 2), cutaneous-muscular (*n* = 1) and disseminated (*n* = 1). There were seven cases of HSA that were divided into primary tumor and secondary (metastatic) tumor because both samples were analyzed. VH were located in encephalon (*n* = 2), scrotum (*n* = 2), testicle (*n* = 1) and periosteum (*n* = 1). HA were cutaneous (*n* = 5), subcutaneous (*n* = 2) and epithelioid (*n* = 1).

### ADRB1, ADRB2 and ADRB3 expression in HA, HSA and VH

**Fig. 1:**
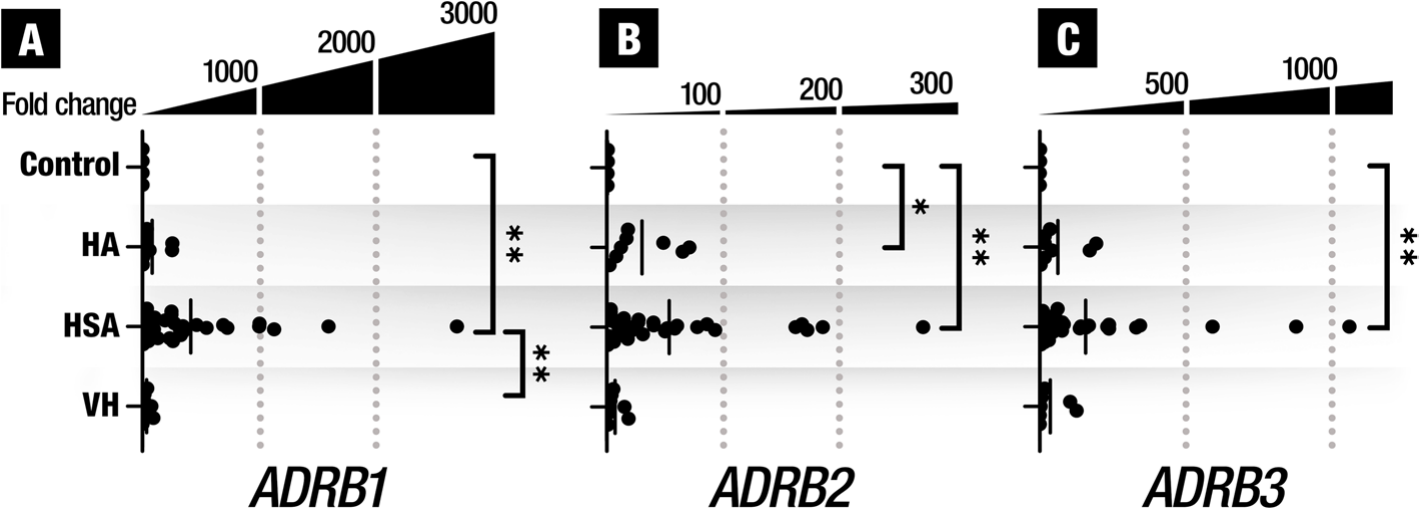
Gene expression of the 3 β-adrenergic receptors subtypes in tissues of control animals and animals presenting hemangiomas (HA), hemangiosarcomas (HSA) and vascular hamartomas (VH). * (*p* < 0.05) and ** (*p* < 0.001) means significant different between groups

Figure 1 shows the mRNA levels of the 3 *ADRB* in HA, HSA and VH. *ADRB1* (*p* = 0.0011) and *ADRB3* (*p* = 0.0046) were overexpressed in HSA groups when compared to the control group, while *ADRB2* was overexpressed in HA (*p* = 0.0305) and HSA (*p* = 0.0020) when compared to the control group. HSA express statistically significantly higher values of *ADBR1* (*p* = 0.0178) compared to VH. Note that there is a high inter-individual variability in the expression of the three subtypes of *ADBR*. However, the levels of gene expression of the three *ADRB* show a clear tendency to increase with the malignancy of the tumor.

### ADRB1, ADRB2 and ADRB3 gene expression in primary and secondary HSA

**Fig. 2:**
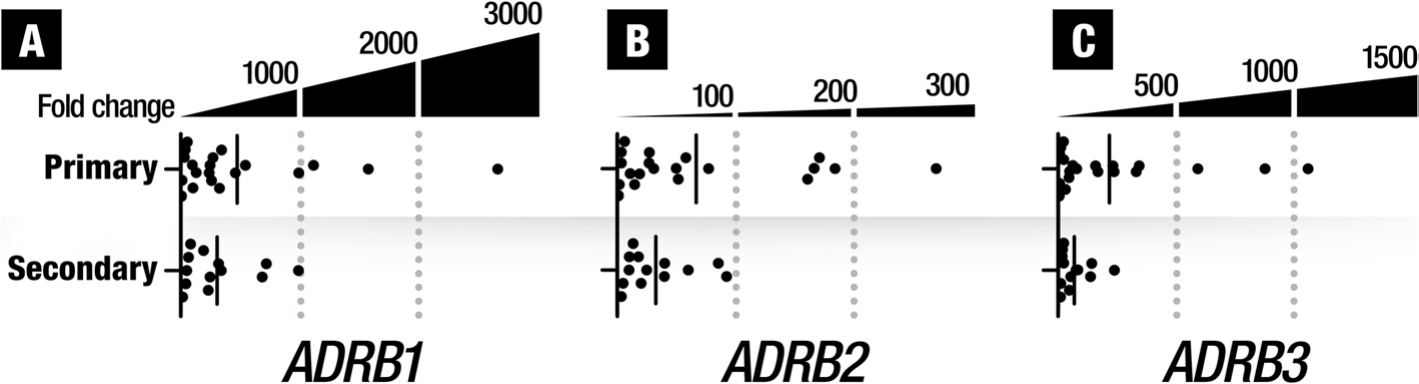
Gene expression of the 3 β-adrenergic receptors subtypes in primary and secondary (metastatic) hemangiosarcoma (HSA). No statistically significant differences were found

We also studied if the expression levels of the three *ADRB* subtypes was different in primary HSA and secondary (metastatic) HSA. We did not find statistically significant differences in the expression levels of the *ADRB1* (*p* = 0,6761), *ADRB2* (*p* = 0,5244) and *ADRB3* (*p* = 0,3157) subtypes between primary and metastatic tumors (Fig. 2).

### ADRB1, ADRB2 and ADRB3 gene expression depending on the tissue localization of the HSA

**Fig. 3:**
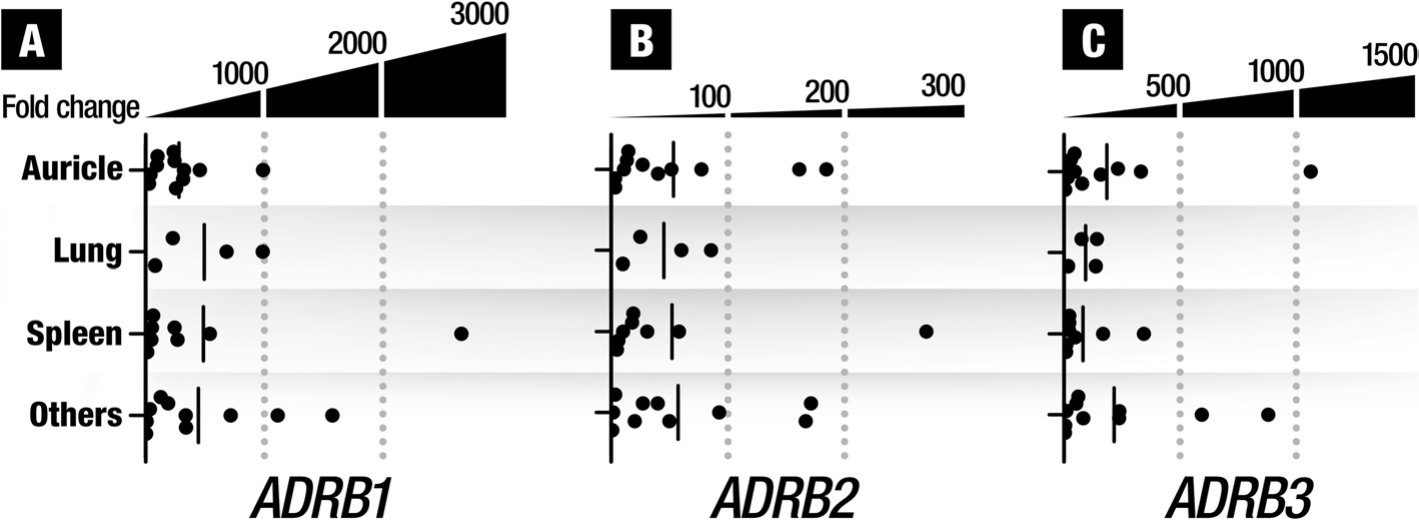
Gene expression of the 3 β-adrenergic receptors subtypes in hemangiosarcoma (HSA) at different localizations. No statistically significant differences were found

We also studied whether the expression levels of the three *ADBR* changed with the HSA tumor localization. We did not find statistically significant differences in the expression levels of any of the three *ADRB* subtypes: *ADRB1* (*p* = 0.8803), *ADRB2* (*p* = 0,0.9520), *ADRB3* (*p* = 0.7744) when comparing among them (Fig. 3).

## Discussion

The canine samples used in the present study come from a population of animals similar in age and breeds described in other studies of canine HSA, HA and VA [10, 16–18, 22–25]. Also, the localization of the primary tumor and the metastatic diseases is in concordance with previous studies and the described progression of canine HSA [10, 17, 18, 22, 26]. Thus, conclusion obtained in our study represent the clinical situation of most vascular tumors and malformations observed in veterinary medicine.

In humans, the three subtypes of β-AR have been identified in HA [6] and HSA [6, 13] among other types of cancer [4]. β1-AR overexpression has been demonstrated in human HSA [9, 13], while β2-AR overexpression is variable [9]. Another study affirms that β2-AR is highly overexpressed in human and canine HSA cell lines [27]. β3-AR is poorly expressed in human HSA, unlike canine HSA cell lines, which predominantly overexpress β2-AR and β3-AR [27]. In a more recent study on 10 samples from visceral HSA and 5 non-malignant splenic hematomas, HSA appeared to express higher levels of immunohistochemistry reactivity towards all three β-AR when compared to the non-malignant hematoma samples. Also, they located the immunoreactivity against β1-AR at the nucleus, β2-AR and β3-AR were mainly observed in the cytoplasm of tumor cells [28]. In the present study we observed that all 3 β-AR were expressed, but the degree of expression was very variable among individual samples. Additionally, we found that they were overexpressed in the primary as well as in secondary or metastatic disease. However, there are more primary samples with clear overexpression of β-AR than those considered secondary. This overexpression could make these samples outliers, the meaning of these outliers is not clear because the overexpression of the β-AR is a characteristic of a more malignant phenotype and initially we hypothesized that metastatic samples may have higher overexpression. Anyway, this observation was not statistically significant and further studies are needed in order to assess any differences in β-AR expression between primary and metastatic HSA in the dog.. A general overview of beta adrenergic signalling and the regulation of tumor cell biology in the context of human and canine HSA have been published [4]. One of the limitations of our study is the lack of follow up for most of the cases studied. It will be interesting to document if β-AR expression is correlated not only with the malignant phenotype but also with prognosis or responds to standard chemotherapy treatment. Another limitation of our study is that we used skin and muscle as control, and they have low expression of β-AR, probably VH could be better control, however, using be this strategy we ensure that we assessed uniformly the β-AR expression in all vascular tumors without losing the data of the VH.

The main interest in the expression of β-AR in tumoral tissues is to evaluate the potential effects of the use of blocker therapy in preventing tumors proliferation [29]. It is clear that in human medicine, propranolol is used as a frontline therapy in the treatment of infantile HA since 2015 [30] and it has been proposed for the treatment of other vascular abnormalities [31]. In the current study, HA significantly overexpressed the *ADBR2* gene (β2-AR), thus it can be hypothesized that a similar clinical situation can be expected to happen in canine patients with HA. However, future prospective studies will be needed to demonstrate that hypothesis.

Propranolol, a β-AR antagonist, has been used with promising outcomes in the treatment of human HSA [4, 9–11]. In our study, and in previous studies with canine HSA cells, it has been demonstrated that all three β-AR are overexpressed [23, 28]. Thus, it can also be hypothesized that its blocking can improve tumor control and prolong survival. Also, a recent study suggests that propranolol synergizes with doxorubicin by a decrease lysosomal sequestration and cellular efflux of doxorubicin while increasing its intracellular concentration and consequently apoptosis [28]. However, as it can be seen in our study results, the expression of β-AR is very variable among individual tumors and the impact of the use of β-AR antagonist needs to be addressed in future prospective studies and in animals were β-AR is documented by inmmunohistochemistry or qPCR in order to obtain adequate data to unravel the β-AR role in canine HSA. Although, there are controversy in human medicine and some reports in human breast cancer and neuroblastoma did not show a clear correlation between β-AR mRNA levels and increased susceptibility to β-AR antagonist therapy [6, 12, 32]. On the other hand, other publications demonstrated high levels of mRNA expression in malignant vascular tumors and, therefore, they suggested a higher sensitivity to β-AR antagonists against these tumors [27]. Also, in human melanoma and colon cancer there are reports pointing a promising use of specific betta blockers in their treatment [29, 33–35]. Recently, the role of β1-AR in an animal model of cavernous vascular malformations and the effect of its specific blocking in preventing these malformations has been studied [36]. In our study, we could not demonstrate a clear overexpression of β-AR receptors in VA and the impact of β-adrenergic blocker in reducing vascular malformations is not clearly predicted as it has been proved in this animal model and human medicine (31).

In conclusion, this study showed overexpression of the three β-AR subtypes in canine HSA and β2-AR in canine HA. High variability was observed in β-AR mRNA levels amongst HSA cases, and we theorize that β-AR antagonists would be useful in dogs with a high β-AR overexpression. Further research is needed to understand the role of β-AR in canine vascular tumors, specially HSA, and the efficacy of β-AR blockers therapy.

## Supplementary Information

**Additional file 1: Table S1.** Summary of canine samples studied with breed, gender, histological diagnosis and tumor localization from where samples were obtained.

## Data Availability

Material available by request to the corresponding authors (josep.pastor@uab.cat).
