# Supplementary material for: Gene expression profiles of beta-adrenergic receptors in canine vascular tumors: a preliminary study
Source: BMC Vet Res. 2022 May 30;18:206. doi: 10.1186/s12917-022-03317-1 (PMC9150297; doi:10.1186/s12917-022-03317-1)
Supplement: Supplementary file 1 — Additional file 1: Table S1. Summary of canine samples studied with breed, gender, histological diagnosis and tumor localization from where samples were obtained. [file 12917_2022_3317_MOESM1_ESM.docx]

Table S1. Summary of canine samples studied with breed, gender, histological diagnosis and tumor localization from where samples were obtained.

| Sample | Breed | Age (yrs) | Gender | Histological diagnosis | Tumor localization |
| --- | --- | --- | --- | --- | --- |
| 1 | Brittany dog | 2 | F | Vascular hamartoma | Encephalon |
| 2 | Great Dane | 9 | M | Vascular hamartoma | Scrotum |
| 3 | Airedale Terrier | 10 | F | Vascular hamartoma | Right pelvic limb |
| 4 | German Shepherd | Unknown | M | Vascular hamartoma | Testicle |
| 5 | Airedale Terrier | 11 | M | Vascular hamartoma | Skin |
| 6 | Boxer | 9 | M | Vascular hamartoma | Scrotum |
| 7 | Unknown | Unknown | M | Vascular hamartoma | Brain |
| 8 | Boxer | 9 | M | Cutaneous cavernous hemangioma | Skin |
| 9 | Crossbreed | Unknown | M | Epithelioid hemangioma | Nictitating membrane |
| 10 | Boxer | 8 | F | Cutaneous hemangioma | Mammary skin |
| 11 | German Shepherd | 7 | F | Cutaneous hemangioma | Skin (elbow) |
| 12 | Boxer | 6 | F | Cutaneous hemangioma | Skin (right flank) |
| 13 | Crossbreed | 14 | F | Subcutaneous hemangioma | Mammary skin (M4) |
| 14 | Labrador Retriever | 8 | M | Cutaneous hemangioma | Interdigital |
| 15 | Crossbreed | 12 | F | Auricular hemangiosarcoma | Right auricle |
| 16 | Labrador Retriever | 11 | F | Auricular hemangiosarcoma | Right auricle |
| 17 | German Shepherd | Unknown | M | Auricular hemangiosarcoma | Right auricle |
| 18 | German Shepherd | 11 | M | Auricular hemangiosarcoma | Right auricle |
| 19-20 | Crossbreed | 9 | M | Auricular and splenic hemangiosarcoma with metastasis | Primary: auricle and spleen; Secondary: lung, kidney and adrenal gland |
| 21 | German Shepherd | 8 | M | Auricular hemangiosarcoma | Right auricle |
| 22 | Crossbreed | 11 | F | Auricular hemangiosarcoma | Right auricle |
| 23 | WHWT | 11 | M | Cutaneous hemangiosarcoma | Skin |
| 24 | German Rough haired Pointer | 10 | M | Cavernous hemangiosarcoma | Skin |
| 25 | German Shepherd | 8 | M | Cutaneous hemangiosarcoma | Subcutaneous mass |
| 26-28 | German Shepherd | 11 | F | Splenic hemangiosarcoma with metastasis | Primary: spleen, Secondary: liver, lung and auricle |
| 29-34 | Cocker Spaniel | 11 | M | Auricular hemangiosarcoma with metastasis | Primary: auricle; Secondary: lung, kidney, spleen, skin and liver |
| 35 | Collie | 3 | M | Cutaneous-muscular hemangiosarcoma | Scapular muscle |
| 36-37 | Golden Retriever | 14 | M | Auricular hemangiosarcoma with metastasis | Primary: auricle; Secondary: liver, lung and kidney |
| 38 | Belgian Shepherd | 10 | M | Cutaneous hemangiosarcoma | Skin |
| 39 | Pitbull | 13 | M | Cutaneous hemangiosarcoma | Skin |
| 40 | Crossbreed | 10 | F | Cutaneous hemangiosarcoma | Skin |
| 41-42 | Crossbreed | 11 | F | Splenic hemangiosarcoma | Primary: spleen; Secondary: auricle |
| 43 | German Shepherd | 9 | M | Splenic hemangiosarcoma | Spleen |
| 44-45 | Crossbreed | 3 | M | Splenic hemangiosarcoma | Primary: spleen; Secondary: auricle |
| 46 | German Shepherd | 13 | M | hemangiosarcoma with metastasis | Spleen, auricular, liver, lung and mesentery |
| 47-48 | German Shepherd | 5 | M | Auricular and splenic hemangiosarcoma | Primary: spleen and auricle; Secondary: lung, kidney and peritoneal and mediastinum fat |
| 49 | French Bulldog | 7 | F | Splenic hemangiosarcoma | Spleen |
| 50 | Warren Hound | 12 | F | Subcutaneous cavernous hemangioma | Subcutaneous |
